# Computed Tomography Features of Gastric Cancer Patients With DNA Mismatch Repair Deficiency

**DOI:** 10.3389/fonc.2021.619439

**Published:** 2021-03-18

**Authors:** Qian Cao, Sheng-Yuan Lai, Nan Xu, Yang Lu, Shuai Chen, Xin-Sheng Zhang, Xiang Li

**Affiliations:** ^1^Radiology Department, Second Affiliated Hospital of Dalian Medical University, Dalian, China; ^2^General Surgery Department, Second Affiliated Hospital of Dalian Medical University, Dalian, China

**Keywords:** gastric cancer, computed tomography, mismatch repair deficiency, microsatellite instability, prognosis

## Abstract

**Objective:**

To explore the computed tomography (CT) features of gastric cancer (GC) patients with DNA mismatch repair deficiency (dMMR).

**Materials and Methods:**

This study reviewed the clinical and CT features of GC patients with dMMR, confirmed by the postoperative results, between September 2017 and December 2019. The expression pattern of MMR major proteins (MLH1, MSH2, MSH6, and PMS2) in immunohistochemistry was used to confirm the MMR status in GC tissues. The correlation between pre-treatment CT features and MMR status was statistically analyzed.

**Results:**

A total of 28 patients with GC were diagnosed as dMMR in our study, and 49 patients were MMR-proficient (pMMR). The tumor locations were significantly different between the dMMR and pMMR groups (p = 0.006). The CT tumor thickness, CT long and short diameters of the largest lymph node, and the number of lymph nodes on CT of the dMMR group were significantly different from the pMMR group.

**Conclusion:**

The dMMR GC exhibited a lower stomach location, smaller tumor thickness and lymph node diameter, and fewer lymph nodes on CT imaging.

## Introduction

Gastric cancer (GC) or adenocarcinoma is one of the most common cancers and a common cause of cancer-related deaths worldwide (1, 2). GC is an aggressive disease, and many GC patients have locally advanced disease at presentation in China (3). The Cancer Genome Atlas has identified microsatellite instability (MSI) with or without DNA mismatch repair deficiency (dMMR) as a hallmark of the second molecular subtype of GC. Immunotherapy in solid malignant tumors, including GC, has been rapidly evolving. Immune checkpoint inhibitors, including anti-programmed death-1 (PD-1) and anti–cytotoxic T-lymphocyte-associated protein-4 (CTLA-4) antibodies, were effective for MSI-high or dMMR solid tumors in many trials (4). However, the dMMR status often requires postoperative pathological immunohistochemical results or polymerase chain reaction testing.

Multi-detector computed tomography (CT) is currently the routine modality of choice for preoperative examination of GC (5–10). CT can provide morphological information about primary tumors, lymph nodes, and suspected distant metastases. A previous report found that dMMR GC features included intestinal-type histology, antral location, and good prognosis with a low rate of recurrence (11). However, whether GC with dMMR has characteristic imaging findings on CT is unknown. In our study, we explored GC patients’ CT features with dMMR for early and advanced GC.

## Materials and Methods

### Patients

This retrospective study was approved by the review board of our institution. The requirement for informed consent was waived. We collected the clinicopathological data of patients with pathologically confirmed GC who underwent radical gastrectomy between September 2017 and December 2019 in our hospital. Some patients underwent two to three cycles of neoadjuvant chemotherapy. All patients underwent baseline contrast-enhanced CT examination of the abdomen. Patients were excluded if they met any of the following criteria: (a) They were detected with distant metastasis in the preoperative examination or during the operation. (b) Patients with poor quality CT images or those who did not undergo preoperative CT due to poor physical condition or other reasons.

**Figure 1:**
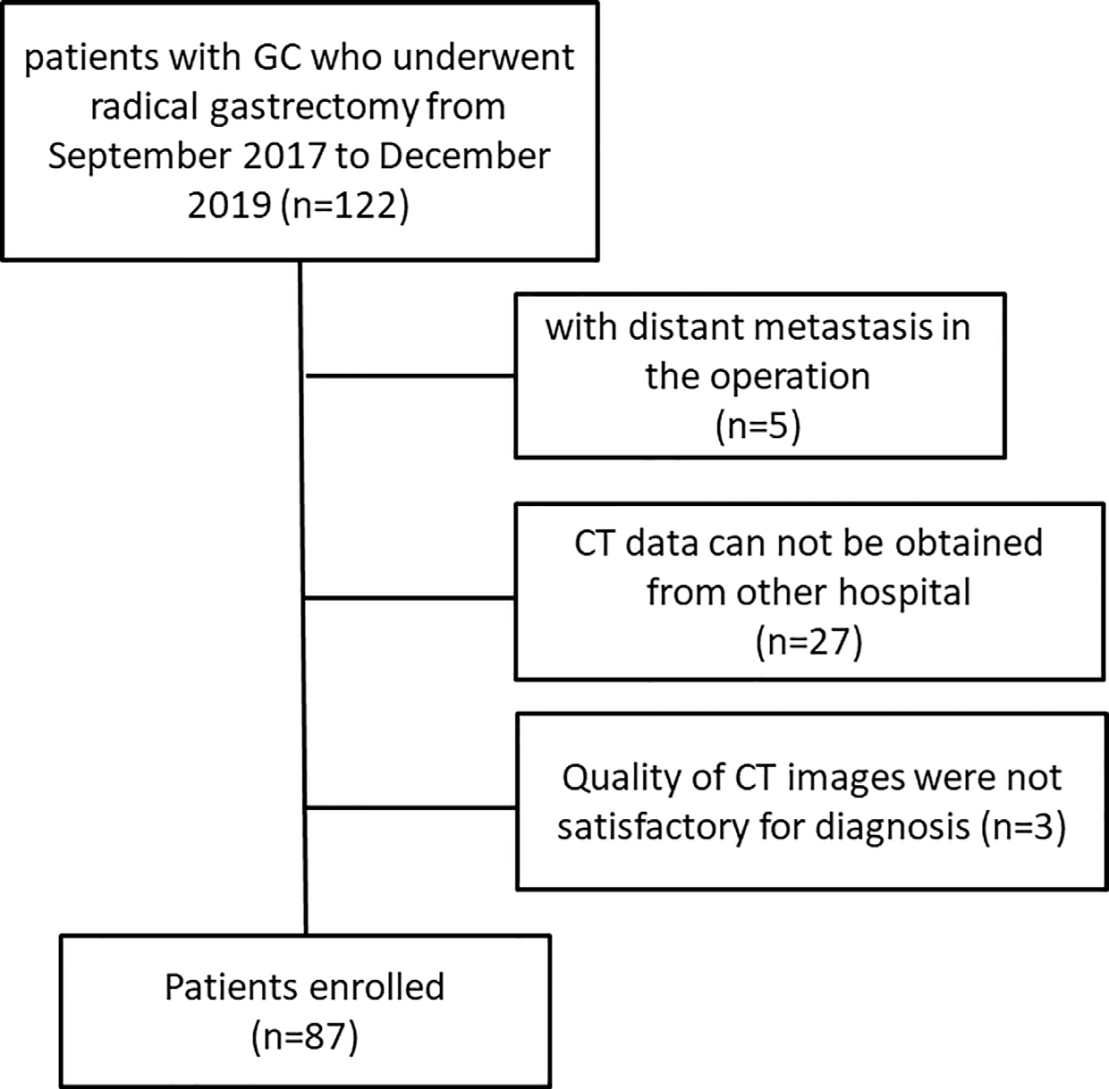
Flowchart of the study design.

Finally, 87 patients (65 males, 22 females, mean age, 58 years; range, 39–85 years) were included in our study, of which 39 patients received radical gastrectomy directly, and 48 patients underwent radical gastrectomy after neoadjuvant chemotherapy (NAC). A flowchart of the study design is presented in **Figure 1**.

### CT Protocol

A CT scanner with 128 rows of detectors was used (Philips Brilliance iCT 256, Royal Dutch Philips Electronics Ltd, Amsterdam, Netherlands). After at least 8 hours of fasting, the patients were given 1,000 ml water for achieving gastric distension. First, the non-contrast CT scan from the diaphragm’s dome to 2 cm below the lower margin of the air-distended gastric body was acquired (collimation: 0.625 mm, peak tube voltage: 120 kVp, tube current-time product: automatic). Next, 100 ml of non-ionic contrast medium (Ultravist, 370 mg/ml; Bayer, Germany) was intravenously administered at 3 ml/s using an automatic injector. Contrast-enhanced CT scans were performed in the arterial phase (30 s) and the portal venous phase (70 s). The portal venous phase was used to evaluate lymph node status. The portal venous phase axial CT images were reconstructed with a 5-mm section thickness and a 5-mm reconstruction interval for clinical interpretation, with a 0.625-mm section thickness for multi planar reformation (MPR) reconstruction.

### Image Analysis

The image analysis was jointly performed by two radiologists with more than 10 years of experience, using the PACS workstation on the axial arterial phase and portal phase CT images. Any discrepancies were resolved by consulting another radiologist with 20 years of experience, and the consensus was achieved. The CT features of GC observed and measured were as follows:

Tumor Location: The location of GC in the CT images was determined by the radiologists, including esophagogastric junction, upper stomach, middle stomach, and lower stomach. We have made the correlation between CT images and endoscopy results to confirm the tumor’s location for every case.

Tumor Thickness: The thickest diameter of the gastric tumor on the axial CT image was measured before and after NAC.

CT Attenuation of Gastric Tumor in Arterial and Portal Phases: The region of interest (ROI) was placed in the whole tumor center with a diameter ≥5 mm. The CT attenuation values of the gastric tumor in the same portion of the axial arterial and portal phase CT images were measured.

Long and Short Diameters of the Largest Lymph Node: The largest regional lymph node’s long and short diameters were measured on axial CT images.

CT Attenuation of the Largest Lymph Node: The CT attenuation values with an oval ROI of the largest regional lymph node on the axial portal phase CT image was measured.

The Number of Lymph Nodes: The number of all the short diameters of gastric regional lymph nodes >5 mm in the axial portal phase images were counted.

### Pathological Diagnosis

The postoperative histopathological diagnosis was performed by an experienced pathologist. The tumor in the gross specimen, the histopathological Lauren classification, and the pathological stage was evaluated based on the eighth AJCC Cancer Staging Manual (12). The expression patterns of MMR major proteins (MLH1, MSH2, MSH6, and PMS2) in immunohistochemistry were used to confirm the MMR status in GC tissues by the experienced pathologist. The lack of expression of any of the four MMR proteins was defined as dMMR. Tumors with the preserved expression of all MMR proteins were considered MMR-proficient (pMMR).

### Statistical Analysis

The continuous and categorical data were presented as mean ± standard deviation and frequency (%). Data processing and analysis were performed using SPSS/PC+ version 22.0 (SPSS Inc, Chicago, IL, USA). The CT features of the dMMR and pMMR groups were compared using the independent-samples t-test and Mann-Whitney U test. A p-value <0.05 was considered statistically significant.

## Results

### Patient and Tumor Characteristics

Eighty-seven patients were included in this study. The patient and tumor characteristics are summarized in **Table 1**. Among the patients, 48 received NAC before surgery. The NAC regimens included SOX (S-1 + oxaliplatin), XELOX (oxaliplatin + capecitabine), and mFOLFOX7 (modified regimen of leucovorin, fluorouracil, and oxaliplatin). The remaining 39 patients underwent surgery without NAC. The tumor locations were significantly different between the dMMR and pMMR groups (p =0.006). The age, gender, tumor size, histological differentiation degree, and pathological stage showed no statistical differences between the dMMR and pMMR groups (p > 0.05) (**Table 1**).

**Table 1:** Patient characteristics.

Clinicopathological features	dMMR (n = 28)	pMMR (n = 59)	p-value
Mean age (range) (years)	60.57 ± 9.87	63.14 ± 8.53	0.217
Male:female	17:11	48:11	0.063
Tumor location			0.006
Esophago-gastric junction	5	34	
Upper stomach	2	3	
Middle stomach	6	7	
Lower stomach	15	15	
Histological differentiation degree			0.106
Low differentiated adenocarcinoma	23	35	
Medium differentiated adenocarcinoma	4	21	
Mucinous adenocarcinoma	0	1	
Neuroendocrine carcinoma	1	2	
Pathological stage without NAC			0.495
IA	3	4	
IB	3	1	
IIA	4	3	
IIA	1	5	
IIIA	4	3	
IIIB	3	5	
Pathological stage after NAC			0.074
IA	0	3	
IB	2	2	
IIA	6	7	
IIB	1	11	
IIIA	1	7	
IIIB	0	5	
IIIC	0	3	
Tumor long size in the gross specimen (cm)	4.26 ± 2.42	4.09 ± 2.40	0.762

*NAC, neoadjuvant chemotherapy.

### Comparison of CT Features Between the dMMR and pMMR Groups of GC

Univariate analysis showed that several CT features were significantly different between the dMMR and pMMR groups during surgery. The CT tumor thicknesses of the dMMR group (11.89 ± 4.87 mm) were less than the pMMR group (14.41 ± 4.70 mm) (p = 0.024). The CT long diameters of the largest lymph node of the dMMR group (8.71 ± 2.43 mm) were less than the pMMR group (10.61 ± 3.82 mm) (p = 0.018). The CT short diameters of the largest lymph node of the dMMR group (6.21 ± 2.17 mm) were less than the pMMR group (7.44 ± 2.85 mm) (p = 0.047). The mean number of lymph nodes on CT of the dMMR group (1.71 ± 1.41) was less than the pMMR group (2.56 ± 1.98 mm) (p = 0.046) (**Table 2**). The CT attenuation of the gastric tumor and the largest lymph node after enhancement showed no significant differences between the dMMR and pMMR patients (**Table 2**).

**Table 2:** Comparison of CT image features between dMMR and pMMR patients.

CT features*	dMMR (n = 28)	pMMR (n = 59)	T	p-value
Tumor thickness (mm)	11.89 ± 4.87	14.41 ± 4.70	2.302	0.024
CT value of gastric tumor in AP (HU)	70.78 ± 27.86	69.64 ± 20.57	0.215	0.083
CT value of gastric tumor in PP (HU)	81.78 ± 21.71	84.25 ± 24.64	0.415	0.679
Long diameters of the largest LN (mm)	8.71 ± 2.43	10.61 ± 3.82	2.402	0.018
Short diameters of the largest LN (mm)	6.21 ± 2.17	7.44 ± 2.85	2.013	0.047
CT value of the largest LN in PP (HU)	60.57 ± 34.06	59.41 ± 28.08	0.169	0.867
Number of lymph nodes on CT	1.71 ± 1.41	2.56 ± 1.98	2.021	0.046

*AP, arterial phase; PP, portal phase; LN, lymph node.

## Discussion

A recent study found that anti–PD-1 therapy with pembrolizumab was clinically beneficial in patients with previously treated unresectable or metastatic MSI-H/dMMR non-colorectal cancer (13). In 2017, the Food And Drug Administration of the United States approved pembrolizumab to treat patients with dMMR/MSI-H non-resectable or solid metastatic tumors. The MSI status is currently used as a biomarker for cancer immunotherapy (14). In our study, we examined some common CT features of the primary tumor and lymph nodes in patients with MSI-H/dMMR of GC.

**Figure 2:**
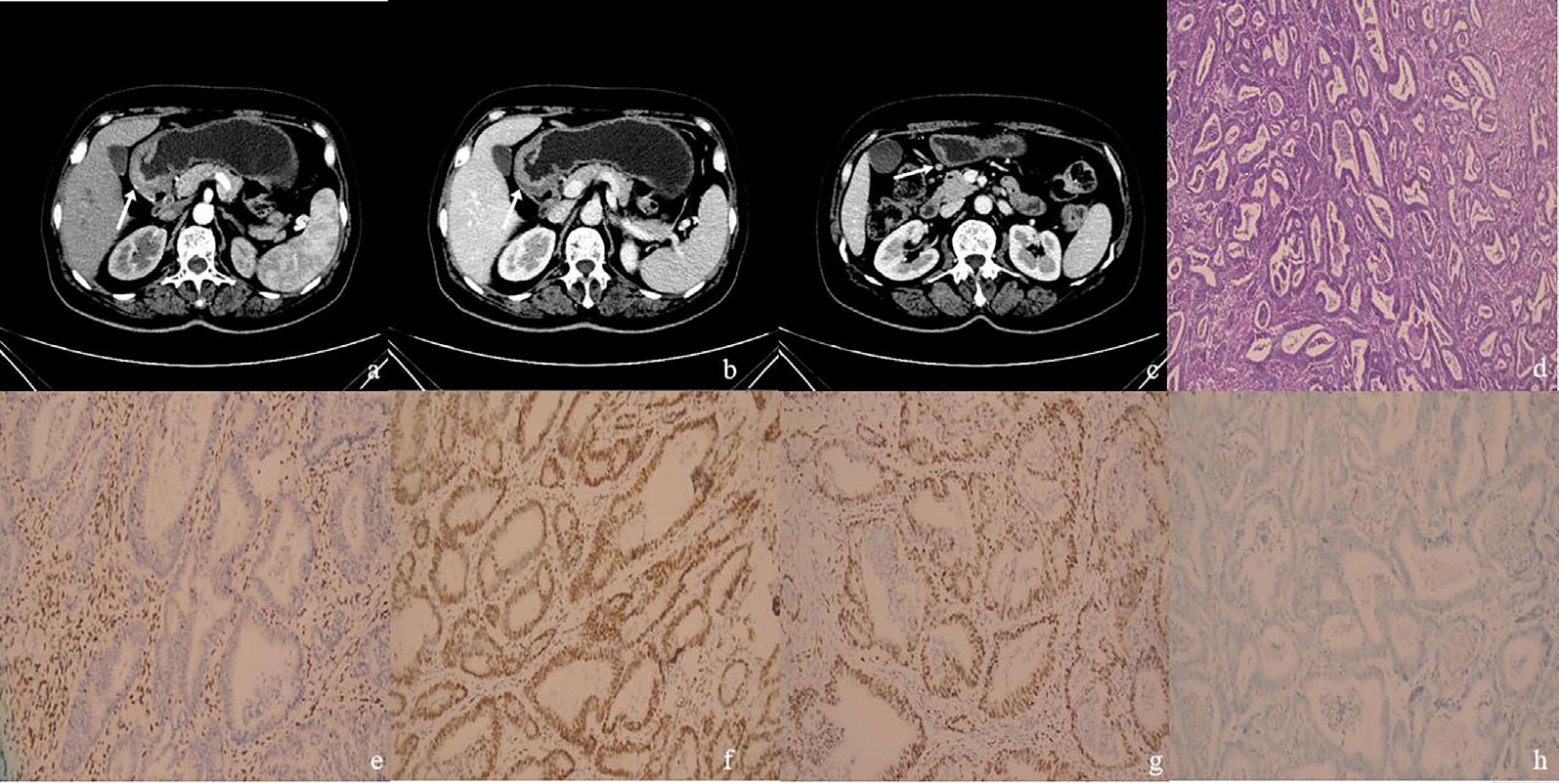
**(A–H)**: One MSI-H&dMMR GC case. Female patient, 64 years old. The postoperative pathologic results showed moderately differentiated adenocarcinoma in gastric antrum with stage T3N0M0, with no metastatic carcinoma in lymph nodes. **(A)** The arterial phase CT value in the enhanced arterial phase of the thickened gastric wall (arrow) in the gastric antrum was 48 HU. **(B)** The portal phase CT value of the thickened gastric wall (arrow) was 67 HU. **(C)** There was a slightly enlarged lymph node (arrow) in No. 4d group around the stomach, with a short diameter of 5 mm and CT value of 72 HU on the portal phase. **(D)** The case of histological analyses by HE staining. **(E–H)** The patient’s immunohistochemical results showed MLH1-negative **(E)**, MSH2-positive **(F)**, MSH6-positive **(G)**, PMS2-negative **(H)**, MSI-H&dMMR.

**Figure 3:**
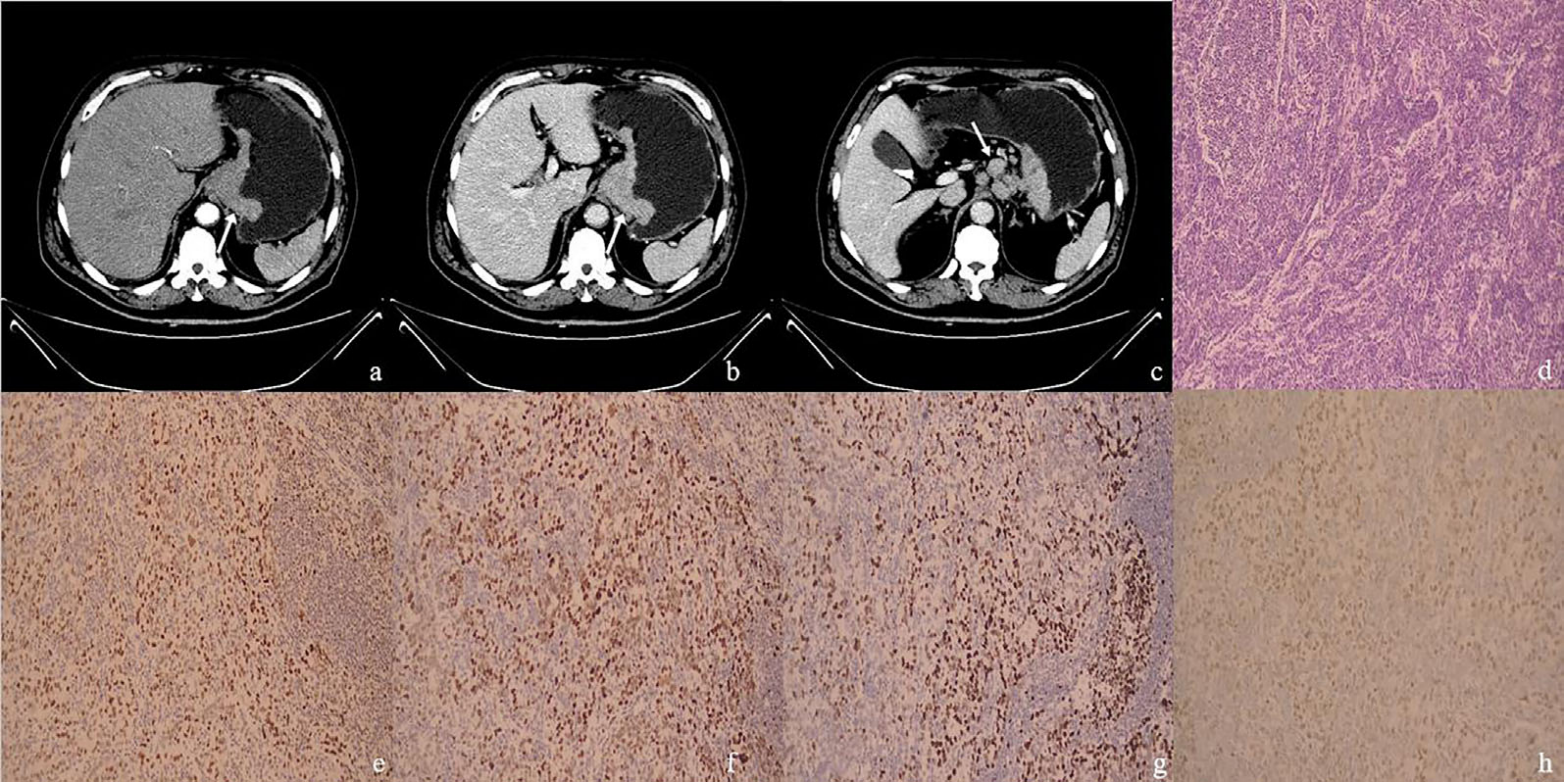
**(A–H)** One MSS&pMMR GC case. Male, 66 years old, surgical pathology results revealed poorly differentiated adenocarcinoma in the gastroesophageal junction, staging T4aN2M0, with metastatic carcinoma in lymph nodes. **(A)** The arterial phase CT value of the mass (arrow) in the gastroesophageal junction was 52 HU; **(B)** The portal phase CT value of the mass (arrow) was 82 HU; **(C)** Multiple enlarged lymph nodes could be seen in the stomach’s lesser curvature. The largest one had a short diameter of 15 mm (arrow), with the CT value of 88 HU on the portal phase. **(D)** The case of histological analyses by HE staining. **(E–H)** The immunohistochemical results showed MLH1-positive **(E)**, MSH2-positive **(F)**, MSH6-positive **(G)**, PMS2-positive **(H)**, MSS, pMMR.

Cristescu R et al. reported that dMMR GC typically has an antral location (11). The results of the present study showed that the tumor locations were significantly different between the dMMR group and the pMMR group. In our study, 53.5% (15/28) dMMR patients were located at the lower stomach. Meanwhile, the main location of the pMMR group was the esophagogastric junction (57.6%, 34/59) (**Figures 2**, **3**). Good prognosis and low recurrence rate were thought to be more common in patients with dMMR GC. Although it has been recognized that the pathological stage was related to prognosis, the tumor size and pathological stage showed no statistical differences between the dMMR and pMMR GC patients in our study.

Given that stomach is a hollow organ, the evaluation of tumor size in the stomach is often influenced by gastric peristalsis. Since the most common gross morphological type of advanced GC was the infiltrating ulcerative type, the boundaries of the tumor on CT images were difficult to identify clearly. In our study, each patient was given 1,000 ml of water to achieve gastric distension. The tumor’s thickness on CT seemed more suitable for evaluating tumor size. The CT tumor thickness of the dMMR group was less than the pMMR group. The CT long and short diameters of the largest lymph node of the dMMR group was less than the pMMR group in our study. Fukuya T et al. found that CT attenuation and lymph-node short-long size ratio could aid in the diagnosis of malignant adenopathy (15). Park et al. considered lymph nodes to be metastatic if the longest diameter was >1.0 cm or if the size was between 0.7 and 1.0 cm with hyper-enhancement, a round shape, central necrosis, or perinodal infiltration (16). We thought that smaller diameters of the lymph node in the dMMR group indicated fewer lymph node metastases and a better prognosis. XP Zhang et al. reported that the number of lymph nodes detected by MDCT showed a significant difference between the lymph node metastasis group and no metastasis group in GC (17). The mean number of lymph nodes on CT in the dMMR group was less than the pMMR group in our study. This may suggest a lower probability of lymph node metastasis in the dMMR group.

This study had some limitations. First, it was a retrospective study. The sample size of the study was relatively small. The findings need to be confirmed by large prospective studies in the future. Second, in addition to MMR status, immunotherapy biomarkers of GC, including tumor mutation burden (TMB) and PD-L1 expression, were not analyzed in our study. Simultaneously, there is no confirmed study on whether neoadjuvant chemotherapy might affect patients’ MMR status, which might require further research.

In summary, this study found that the dMMR GC exhibited a lower stomach location, smaller tumor thickness and lymph node diameter, and fewer lymph nodes on CT imaging.

## Data Availability Statement

The raw data supporting the conclusions of this article will be made available by the authors, without undue reservation.

## Ethics Statement

Ethical review and approval was not required for the study on human participants in accordance with the local legislation and institutional requirements. Written informed consent for participation was not required for this study in accordance with the national legislation and the institutional requirements.

## Author Contributions

Conceptualization: X-SZ and XL. Data curation: QC and S-YL. Formal analysis: QC and NX. Funding acquisition: XL. Methodology: QC, YL, and SC. Supervision: X-SZ and XL. Validation: QC, S-YL, and XL. Writing: QC and S-YL. All authors contributed to the article and approved the submitted version.

## Funding

This work was supported by Liaoning Provincial Natural Science Foundation of China (Grant Nos. 2019-ZD-0645).

## Conflict of Interest

The authors declare that the research was conducted in the absence of any commercial or financial relationships that could be construed as a potential conflict of interest.
